# Author's Reply

**DOI:** 10.18502/ijrm.v20i12.12569

**Published:** 2023-01-09

**Authors:** Mohammad Rafiei

##  Author's Reply

Thanks for your observations and for giving us a chance to reply to the letter you received about our paper entitled: “The relationship between body mass index and preeclampsia: A systematic review and meta-analysis”. In the following, the authors have tried to answer the issues raised as much as possible:

The quality assessment was done by the Newcastle-Ottawa Scale, 27 articles with a quality score of < 3 were excluded from the study, and 16 high-quality studies were analyzed (Figure 1). The article was written according to PRISMA guidelines (1). According to these guidelines reporting the quality assessment details is not obligatory in all meta-analysis, in meta-analysis conducted on randomized clinical trials and Cochrane articles, this section is usually reported in analytically in most articles. But only 30% of meta-analysis report quality assessment in the results of articles (Page 9, Table II, Item 22) (2).

Many statistical tests, such as Begg and Mazumdar (1994), can be used to check publication bias. In this article, we report the p-value and a plot in the results section, which seems to be sufficient (Page 469, Table II (p = 0.584), Figure 5). Egger et al. proposed a test for the asymmetry of the funnel plot. The power of this method to detect bias will be low with small numbers of studies. Begg and Mazumdar proposed testing the interdependence of variance and effect size using Kendall's method. This bias indicator makes fewer assumptions than that of Egger et al. (3, 4).

Pubmed and Scopus databases for English and SID for Persian articles had good coverage for our search, no search results for additional articles were found on Web of Sciences. Using the Web of Sciences is not obligatory in search of all meta-analysis studies. In our investigation, there were neither any articles in the Web of Sciences nor in PubMed and Scopus databases.

Subgroup analysis were done according to mild and severe preeclampsia (Figure 3, 4). To pool the results of articles, there were alternative, fixed effect models, (when heterogeneity is not significant) and random effect models (when heterogeneity is significant) (5).

Effect size was defined by the researcher as mean BMI with a 95% confidence interval (CI), when we use CI there is the sample size in its formula (mean $\pm z\sigma /\sqrt{n}$ ).

Effect size can be defined as mean difference, or SMD, WMD, OR, RR, if we use SMD it would lead to an increase in missing values, SMD is computed when mean, SD, and sample size are available in both cases and control groups, but when we define mean BMI as effect size, it is not necessary we have mean, SD, and sample size in both groups.

Consequently, the results of this article, according to pooled results of 16 high-quality articles, are valid to cite.

**Morteza Motedayen^1^ M.D., Mohammad Rafiei^2^ Ph.D., Mostafa Rezaei Tavirani^3^ Ph.D., Kourosh Sayehmiri^4^ Ph.D., Majid Dousti^4^ Ph.D.**

^1^Department of Cardiology, Faculty of Medicine, Zanjan University of Medical Sciences, Zanjan, Iran.

^2^Department of Biostatistics and Epidemiology, School of Medicine, Arak University of Medical Sciences, Arak, Iran.

^3^Proteomics Research Center, Shahid Beheshti University of Medical Sciences, Tehran, Iran.

^4^Psychosocial Injuries Research Center, Department of Biostatistics, School of Public Health, Ilam University of Medical Sciences, Ilam, Iran.
